# Microalgae as Sources of Carotenoids

**DOI:** 10.3390/md9040625

**Published:** 2011-04-20

**Authors:** Ana Catarina Guedes, Helena M. Amaro, Francisco Xavier Malcata

**Affiliations:** 1 CIMAR/CIIMAR—Centro Interdisciplinar de Investigação Marinha e Ambiental, Universidade do Porto, Rua dos Bragas 177, P-4050-123 Porto, Portugal; E-Mails: acatarinaguedes@gmail.com (A.C.G.); lena.amaro@gmail.com (H.M.A.); 2 ISMAI—Instituto Superior da Maia, Avenida Carlos Oliveira Campos, P-4475-690 Avioso S. Pedro, Portugal; 3 Instituto de Tecnologia Química e Biológica, Universidade Nova de Lisboa, Avenida da República, P-2780-157 Oeiras, Portugal

**Keywords:** lutein, astaxanthin, β-carotene, bioproduction, extraction

## Abstract

Marine microalgae constitute a natural source of a variety of drugs for pharmaceutical, food and cosmetic applications—which encompass carotenoids, among others. A growing body of experimental evidence has confirmed that these compounds can play important roles in prevention (and even treatment) of human diseases and health conditions, e.g., cancer, cardiovascular problems, atherosclerosis, rheumatoid arthritis, muscular dystrophy, cataracts and some neurological disorders. The underlying features that may account for such favorable biological activities are their intrinsic antioxidant, anti-inflammatory and antitumoral features. In this invited review, the most important issues regarding synthesis of carotenoids by microalgae are described and discussed—from both physiological and processing points of view. Current gaps of knowledge, as well as technological opportunities in the near future relating to this growing field of interest, are also put forward in a critical manner.

## 1. Introduction

Microalgae occupy the bottom of the food chain in aquatic ecosystems; they possess the intrinsic ability to take up H_2_O and CO_2_—which, with the aid of sunlight, are converted to complex organic compounds that are subsequently kept inside or released from the cell. Those microorganisms have a worldwide distribution, and are well-adapted to survive under a large spectrum of environmental stresses—including (but not limited to) heat, cold, drought, salinity, photo-oxidation, anaerobiosis, osmotic pressure and UV exposure [1].

Microalgae combine, in a balanced way, a few properties typical of higher plants (*viz*. efficient oxygenic photosynthesis and simple nutritional requirements) with biotechnological attributes proper of microorganisms (*viz*. fast growth rates, and ability to accumulate or secrete primary and secondary metabolites). This rather useful combination has led to selection of such microorganisms for applied processes, and represents the basic rationale for the usefulness of microalgal biotechnology. Besides being currently used as feed for aquatic and terrestrial animals, the nutritional value of microalgal biomass goes well beyond—and includes use as colorant in aquaculture, and high-protein or polyunsaturated fatty acid supplement in human diets. The food, pharmaceutical and cosmetic markets have accordingly benefited from a growing array of microalgal products [2,3].

Furthermore, the large number of existing species of microalgae constitutes a unique reservoir of biodiversity, which supports potential commercial exploitation of many novel products besides vitamins, pigments and polyunsaturated fatty acids [4–6]. The key factor for their eventual economic feasibility is the possibility of operating large photobioreactors, able to handle biomass and metabolites to sufficiently high levels [7,8].

This review covers the most relevant features of a family of specialty products originated in microalgae that have already reached commercial expression—by presenting bioprocess considerations and reviewing practical applications, mainly in the food and health industries.

## 2. Cellular Location and Function

Carotenoids constitute a class of terpenoid pigments, derived from a 40-carbon polyene chain, which can be envisaged as their molecular backbone—indeed it provides carotenoids with distinctive molecular structures, and the associated chemical properties including light-absorption features that are essential for photosynthesis and, in general, for life in the presence of oxygen [9]. The aforementioned backbone may be complemented by cyclic groups (rings) and oxygen-containing functional groups. Hence, hydrocarbon carotenoids are denoted as carotenes as a whole, but oxygenated derivatives are known specifically as xanthophylls—with oxygen being present as –OH groups (e.g., lutein), as oxi-groups (e.g., cantaxanthin) or as a combination of both (e.g., astaxanthin) [9]. All xanthophylls synthesized by higher plants—e.g., violaxanthin, antheraxanthin, zeaxanthin, neoxanthin and lutein, can also be synthesized by green microalgae; however, these possess additional xanthophylls, e.g., loroxanthin, astaxanthin and canthaxanthin. Diatoxanthin, diadinoxanthin and fucoxanthin can also be produced by brown algae or diatoms [10].

A distinction is usually made between primary and secondary carotenoids: primary ones (*i.e*., xanthophylls) are structural and functional components of the cellular photosynthetic apparatus, so they are essential for survival [10]; whereas secondary ones encompass those produced by microalgae to large levels, but only after exposure to specific environmental stimuli (via carotenogenesis).

Xanthophylls are relatively hydrophobic molecules, so they are typically associated with membranes and/or involved in non-covalent binding to specific proteins; they are usually localized in the thylakoid membrane, whereas secondary carotenoids are found in lipid vesicles—in either the plastid stroma or the cytosol. Most xanthophylls in cyanobacteria and oxygenic photosynthetic bacteria are associated with chlorophyll-binding polypeptides of the photosynthetic apparatus [11]; however, in most green microalgae, carotenes and xanthophylls are synthesized within plastids, and accumulate therein only. Conversely, secondary xanthophylls in some green microalgae—e.g., astaxanthin in *Haematococcus* sp., accumulate in the cytoplasm; this realization raises the possibility of an extra-plastidic site of carotenoid biosynthesis in that genus. Alternatively, xanthophylls synthesized in the chloroplast may be exported, and consequently accumulate in the cytoplasm [10,12,13]—so they may be found in essentially all cellular compartments.

Carotenoids perform several functions in microalgae: they are involved in light harvesting, but also contribute to stabilize the structure and aid in the function of photosynthetic complexes—besides quenching chlorophyll triplet states, scavenging reactive oxygen species and dissipating excess energy [14]. The intrinsic antioxidant activity of carotenoids constitutes the basis for their protective action against oxidative stress; however, not all biological activities claimed for carotenoids relate to their ability to inactivate free radicals and reactive oxygen species.

## 3. Practical Applications

Several researchers have actively focused on carotenoids from microalgal sources; the major areas, in terms of actual or potential industrial applications, are food and health—and the antioxidant properties exhibited by that class of compounds constitutes at present its core interest. Pigments of microalgal origin are indeed experiencing a strong market demand: the price of microalgal β-carotene easily attains 700 €/kg, whereas its synthetic counterpart cannot reach more than half that figure. Natural β-carotene is preferred by the health market because it is a mixture of *trans* and *cis* isomers—the latter of which possess anticancer features; such a mixture can hardly be obtained via chemical synthesis [14].

### 3.1. Uses for Food and Feed Formulation

Manufacture of carotenoids via microbiological routes has undergone a greater and greater scientific and commercial importance within the alimentary and aquaculture fields [15], especially in view of environmental and health awareness by consumers at large.

Recall that most oxidation reactions in foods are deleterious—e.g., degradation of vitamins, pigments and lipids, with consequent loss of nutritional value and development of off-flavors [16,17]. Antioxidants—which are adventitious in, or deliberately added to foods, can inhibit oxidation or slow down initiation by free alkyl radicals, as well as interrupt propagation of such free radical chains. The threshold of synthetic food additives legally permitted has been steadily decreasing, due to their suspected role as promoters of carcinogenesis, besides claims of liver and renal toxicities [18]; hence, substitution thereof by natural pigments has become common practice. One good example is the application of *Dunaliella* spp. for mass production of carotenoids aimed at a preservation role [19,20]. Another advantage of carotenoids is that they are not affected by the presence of ascorbic acid, often used as acidulant to constrain unwanted microbial growth, nor by heating/freezing cycles employed in foods with a similar goal.

On the other hand, carotenoids are particularly strong dyes, even at levels of parts per million. Specifically, canthaxanthin, astaxanthin and lutein from *Chlorella* have been in regular use as pigments, and have accordingly been included as ingredients of feed for salmonid fish and trout, as well as poultry—to enhance the reddish color of said fish or the yellowish color of egg yolk [4,21–23]. Furthermore, β-carotene has experienced an increasing demand as pro-vitamin A (retinol) in multivitamin preparations; it is usually included in the formulation of healthy foods, although only under antioxidant claims [24–26].

### 3.2. Uses for Health and Well-Being Promotion

In the human being, oxidation reactions driven by reactive oxygen species can lead to protein damage and DNA decay or mutation; these may in turn lead to several syndromes, *viz*. cardiovascular diseases, some kinds of cancer and degenerative diseases, and ageing at large [17,27]. As potent biological antioxidants, carotenoids are able to absorb the excitation energy of singlet oxygen radicals into their complex ringed chain—thus promoting energy dissipation, while protecting tissues from chemical damage. They can also delay propagation of such chain reactions as those initiated by degradation of polyunsaturated fatty acids—which are known to dramatically contribute to the decay of lipid membranes, thus seriously hampering cell integrity [21].

One illustrative example is the decline of cognitive ability accompanying Alzheimer’s disease, which is apparently caused by persistent oxidative stress in the brain [28]. Using transgenic mice fed with extracts from *Chlorella* sp. containing β-carotene and lutein, Nakashima *et al.* [29] claimed significant prevention of cognitive impairment. Wu *et al*. [30] used also *Chlorella* extracts containing 2–4 mg/g_DW_ of lutein, and reported reduction in the incidence of cancer, as well as prevention of macular degeneration [31]. Likewise, carotenoids extracted specifically from *Chlorella ellipsoidea* and *Chlorella vulgaris* inhibited colon cancer development [23]. Furthermore, astaxanthin obtained from *Haematococcus pluvialis* decreased expression of cyclin D1, but increased that of p53 and some cyclin kinase inhibitors of colon cancer cell lines [32].

Carotenoids have also the ability to stimulate the immune-system, thus being potentially involved in more than 60 life-threatening diseases—including various form of cancer, coronary heart diseases, premature ageing and arthritis [33]; this is specifically the case of canthaxanthin and astaxanthin, and other nonprovitamin A carotenoids from *Chlorella* but to a lesser degree [23]. A few epidemiological studies encompassing β-carotene from *Dunalliela* sp.—which contains readily bioavailable 9-*cis* and all-*trans* stereoisomers (*ca*. 40% and 50%, respectively), have indeed provided evidence of a lower incidence of several types of cancer and degenerative diseases [34]. Finally, carotenoids exhibited hyperlipidemic and hypercholesterolemic effects [19].

## 4. Industrial Production

The worldwide demand for carotenoids was *ca*. 640 M€ in 2004, but it has been rising ever since at an average yearly rate of 2.2% [9]; β-carotene has specifically risen from *ca*. 175 M€ in 2004 to *ca*. 183 M€ in 2009 [35]. A growing fraction has been accounted for by carotenoids from biotechnological sources; and β-carotene, as well as such xanthophylls as astaxanthin, cantaxanthin and lutein have consequently been in higher and higher demand [9]. The most famous source microalgae are *Chlorella*, *Chlamydomonas*, *Dunaliella*, *Muriellopsis* and *Haematococcus* spp.—all of which belong to the Chlorophyceae family [2]. They tend to accumulate carotenoids as an intrinsic part of their biomass, thus offering economical alternatives to chemical synthesis [36].

Among all natural sources studied to date, *Dunaliella* possesses the highest content of 9-*cis* β-carotene [20,34]—reaching levels up to 100 g/kg_DW_, [19,37,38]; β-carotene-rich *Dunaliella* powder has been commercially exploited in many countries since the 1980s. Although many microalgae can produce xanthophylls, *H. pluvialis* is the one that accumulates them to the highest levels (e.g., asthaxanthin [10]), so it is now cultivated at large scale by several companies using distinct approaches [39]. On the other hand, *Muriellopsis* sp. holds a high lutein content (up to 35 mg L^−1^), coupled with a high growth rate; hence, it has been exploited for commercial production of lutein [10]. Finally, *C. ellipsoidea* was reported to produce violaxanthin, together with two other minor xanthophylls, *viz*. antheraxanthin and zeaxanthin—whereas the main carotenoid in *C. vulgaris* was lutein [23]. Further pieces of related information are gathered in Table 1.

## 5. Biotechnological Processes

Despite a few useful features already referred to above, microalgae are in general expensive to produce, so concerted efforts have been on the way toward more cost-efficient modes of mass cultivation.

With regard to open systems, the best choice seems to be the open shallow pond—made of leveled raceways, 2–10 m wide and 15–30 cm deep, which run as simple loops or meandering pathways; each unit may cover an area of several hundred to a few thousand m^2^. However, this configuration poses several problems—which restrict its use to strains that, in view of their weed-like behavior (e.g., *Chlorella*) or their ability to withstand adverse growing conditions (e.g., *Spirulina* or *Dunaliella*), can outgrow other microorganisms.

More advanced technologies have meanwhile been made available pertaining to closed systems; these provide better options for growth of most microalgal strains, by protecting the culture from contamination by unwanted (and often ill-defined) microorganisms, and allowing comprehensive and integrated control of processing conditions. Such photobioreactors are either flat or tubular, and may adopt a variety of designs and operation modes. They lead to higher volumetric productivities and an overall better quality for the biomass (or product) generated—but they are also more expensive to build and operate than their open counterparts [9].

Some microalgae exhibit unique productivity and plasticity features: when grown under distinct sets of operating conditions, they may accumulate different products to high levels; hence, careful design and control of medium composition, temperature, pH, aeration, stirring and irradiance are recommended. A few examples of optimum conditions of operation of microalgal reactors—using productivity of carotenoids as objective function, are listed in Table 2.

During microalgal cultivation, a few processing parameters can be specifically manipulated for maximum synthesis of carotenoids; the better studied cases are lutein, astaxanthin and β-carotene—which will be discussed below at some length.

### 5.1. Lutein

The most important factors that affect lutein content in microalgae are temperature, irradiance, pH, availability and source of nitrogen, salinity (or ionic strength) and presence of oxidizing substances (or redox potential); however, specific growth rate also plays a crucial role.

High temperature favors accumulation of lutein, as happens with other carotenoids (e.g., β-carotene) in *Dunaliella* sp. [42], close to the limit of environmental stress; further temperature increases would thus be harmful, and eventually reduce biomass productivity.

A high irradiance level appears beneficial—but its effect depends on whether indoor or outdoor cultivation is considered; *in vitro* mimicking of all parameters that characterize outdoor operation, e.g., solar cycle and temperature fluctuation, is indeed difficult. Furthermore, the concentration of molecular oxygen outdoors cannot be manipulated, despite its interacting with illumination and temperature. Both irradiance and temperature influence the rate of lutein production, yet cultures of *Murielopsis* sp. and *Scenedesmus almeriensis* produced contradictory results; hence, these two factors should be considered in a combined, rather than independent fashion [8].

Likewise, the reported effects of pH are not consistent between batch and continuous cultivations. In the former, lutein content increased at extreme pH values, whereas the best results under continuous operation were observed at the optimum pH for growth rate. It is worth noting that pH is particularly relevant in microalgal cultures because it interferes with CO_2_ availability (which is essential for photosynthesis); hence, continuous supply of CO_2_, as a fraction of the aeration stream, and pH-controlled injection lead to different results. In general, the maximum lutein productivity is achieved at the optimum pH for biomass productivity [45].

The concentration of nitrogen in the culture medium (in the form of nitrate) does not apparently cause a significant effect upon the lutein content of biomass; however, n-limitation reduces biomass productivity, and consequently leads to poor overall lutein synthesis. Hence, nitrate should be supplied to a moderate excess—so that growth rate is not hampered, while avoiding saline stress that dramatically affects culture performance [8].

Lutein synthesis is enhanced via addition of such chemicals as H_2_O_2_ and NaClO, which behave as inducers: in the presence of Fe^2+^, they affect the redox state and generate stress-inducing chemical species. This induction of oxidative stress is expected because lutein holds a protection role conveyed by its antioxidant features—particularly under heterotrophic growth, where spontaneous oxidative stress is normally absent (unlike happens with phototrophic cultures) [45].

Finally, the specific growth rate affects both continuous and semicontinuous cultures: lutein tends to accumulate at low dilution rates, but not to levels sufficient to balance the decrease in biomass productivity under such circumstances. Therefore, the maximum lutein productivity is again typically attained at the optimal dilution rate for biomass production [45].

### 5.2. Astaxanthin

Commercial production of astaxanthin by *Haematococcus* sp. has been implemented by more than one microalga company (e.g., Cyanotech and Aquasearch); they resorted to a two-stage system, consisting of a first step to produce green biomass under optimal growth conditions (“green” stage), followed by a second stage when the microalga is exposed to adverse environmental conditions to induce accumulation of astaxanthin (“red” stage) [50]. Astaxanthin productivities in large scale facilities are typically *ca*. 2.2 mg L^−1^ [39]—even though maximum astaxanthin productivities of 11.5 mg L^−1^ d^−1^ can be attained at bench scale [51].

Micro Gaia, a marine biotech firm engaged in production of microalgae rich in astaxanthin, proposed a single-step, continuous manufacture process using moderate nitrogen limitation [52,53]: the biomass and astaxanthin productivities obtained were 8.0 and 0.7 mg L^−1^d^−1^, respectively [54]. The feasibility of the latter approach for production of astaxanthin by *H. pluvialis* was tested continuous-wise in outdoor apparatuses [48]: Aquasearch Growth Modules (AGM)—*i.e*., 25,000 L enclosed, computerized photobioreactors, were combined up to three units to obtain large amounts of clean, fast growing *H. pluvialis*; they were transferred daily to a pond culture system, where carotenogenesis and astaxanthin accumulation were induced. After 5 days of synthesis, cells were harvested by gravitational settling—with a typical content of 2.5% (w/w_DW_) astaxanthin; a high pressure homogenizer was used to disrupt the cells, and then drying was carried out to less than 5% (w/w) moisture. The performance of AGM could be improved 2-fold within the first 9 mo of operation; and the biomass concentration increased from 50 to 90 g m^−2^, with associated productivities increasing from 9 to 13 g m^−2^ d^−1^ within the same period [39].

However, the production capacity of *H. pluvialis* was constrained by its intrinsic slow growth, low cell yield, ease of contamination by bacteria and protozoa, and susceptibility to adverse weather conditions [5]. Moreover, *H. pluvialis* cannot be efficiently cultivated in dark heterotrophic mode—so production of astaxanthin should adopt the photosynthetic mode, and thus resort to levels of irradiance (e.g., 950 μmol m^−2^ s^−1^) well beyond what would be economically reasonable [39]. Owing to its ease of culturing and high tolerance to environmental fluctuations, *C. zofingiensis* (another green microalga) has been put forward as an alternative for astaxanthin production: it grows quite fast (*ca*. three times faster than *H. pluvialis*), and accumulates significant amounts of secondary carotenoids in the dark, thus facilitating large-scale cultivation of denser biomass [47,55].

Oxidative stress induced by intense illumination has been found to play a crucial role upon astaxanthin synthesis [56]; active oxygen molecules, generated by excess photooxidation caused by high light irradiance, do apparently trigger synthesis of carotenoids as part of a cellular strategy aimed at cell protection against oxidative damage [47]. In particular, flashing light increased the rate of astaxanthin production per photon in *H. pluvialis* by at least 4-fold relative to that under continuous light sources [57]—thus proving that light quality is more important than quantity [58].

The effect of irradiance depends also on such operating variables as culture density, cell maturity (flagellates are much more sensitive than palmelloids), medium nutrient profile and light path [59]. The predominant role of light stress and nitrogen deprivation towards induction and enhancement of biosynthesis in the aplanospores of *H. pluvialis* was originally suggested in the 1950s [60]; astaxanthin accumulation comes along with growth halting, as happens in most cases of stress imposed upon microalgae [59,61]. Imamoglu *et al.* [54] compared the effect of various stress media, under high light intensities, upon astaxanthin accumulation; those authors concluded that addition of CO_2_ in an N-free medium, under 546 μmol_photon_ m^−2^ s^−1^, were the best conditions for accumulation of astaxanthin—which attained *ca*. 30 mg g^−1^.

Astaxanthin may thus be efficiently produced outdoors in continuous mode, if accurate nitrate dosage is provided [48]; besides N, such oligoelements as iron play a role. This essential oligoelement takes part in assimilation of nitrate and nitrite, deoxidation of sulphate, fixation of N, and synthesis of chlorophyll [62–65]. Iron deficiency was reported to constrain microalga growth, even in rich nutrient media [64]; whereas its addition enhanced astaxanthin synthesis [66–69]. Cai *et al*. [67] further tested how iron electrovalencies and counter ions affect cell growth and accumulation of astaxanthin; 18 μmol L^−1^ Fe^2+^-EDTA stimulated synthesis of astaxanthin more effectively, up to contents of 30.7 mg g^−1^; and despite the lower cell density attained (2.3 × 10^5^ cell mL^−1^), a higher concentration (36 μmol L^−1^) of FeC_6_H_5_O_7_ yielded cell density and astaxanthin production levels that were 2- and 7-fold those reached under iron-limitation.

In the “red stage” of growth, *Haematococcus* cells require only carbon as major nutrient—which this is usually supplied via directly injecting CO_2_ into the photobioreactor during daylight [61]. Furthermore, high irradiance provides more energy for photosynthetic fixation of C, which leads to a higher rate of astaxanthin synthesis [68]; this may be further enhanced by raising the C/N ratio [69].

Finally, Chen *et al.* [70] experimented with heterotrophic conditions—using pyruvate, citrate and malate as substrates, towards synthesis of astaxanthin by *C. zofingiensis* in the absence of light. Presence of any of the aforementioned substrates above 10 mM stimulated biosynthesis of astaxanthin (and other secondary carotenoids); *ca*. 100 mM pyruvate led to yields of 8.4–10.7 mg L^−1^ astaxanthin, which correspond to a 28%-increase.

### 5.3. β-Carotene

Semicontinuous cultivation of *D. salina* at 25 °C produced 80 g m^−3^ d^−1^ biomass [42]—from which 1.25 mg L^−1^ of β-carotene was recovered [71]; however, this figure could be improved up to 2.45 mg m^−3^ d^−1^ in continuous biphasic bioreactors [72]. When cultivated photoheterotrophically, a significant increase of cellular β-carotene content was experimentally observed: the maximum score was 70 pg cell^−1^, in a culture enriched with 67.5 mM acetate and 450 μM FeSO_4_ [33].

As with astaxanthin, Fe^2+^ plays an important role in β-carotene accumulation in *D. salina*; by inducing oxidative stress, those cations stimulate said synthesis, especially in the presence of a carbon source. UV-A radiation (320–400 nm) added to the photosynthetically active radiation (PAR, *i.e*., 400–700 nm) can be regarded as another stress factor during growth of, and carotenoid accumulation by *Dunalliela bardawil;* compared with cultures exposed to PAR only, addition of 8.7 W m^−2^ UV-A radiation to 250 W m^−2^ PAR stimulated long-term growth of that microalga, and led to a 2-fold enhancement in β-carotene accumulation by 24 d [38].

## 6. Extraction and Purification

Although microalga-mediated synthesis of carotenoids is crucial in biotechnological production thereof, a major portion (if not most) of their cost actually lies on downstream separation—e.g., biomass drying and disruption, followed by solvent extraction and purification. Hence, these issues are addressed below, in view of their importance toward commercial scale processes.

### 6.1. Cell Disruption

A major practical problem in using such microalgae as *Murielopsis* sp. or *S. almeriensis* is the need for cell wall disruption. This can be accomplished through a variety of ways, e.g., milling, ultrasound, microwave, freezing, thawing or chemical attack [45].

The mortar-and-pestle procedure described by Mínguez-Mosquera *et al*. [73] provides full recovery, but it cannot be scaled up to industrial practice; sonication and ball milling produce results similar to that procedure, as long as alumina is employed as disaggregating agent [45]. Ceron *et al*. [74] complemented the alumina-based cell disruption with alkaline treatment using 4% (w/v) aqueous KOH (40 °C); disaggregation and lipid expression were both facilitated.

### 6.2. Biomass Extraction

Microalgal biomass is usually processed via solvent extraction, to render carotenoid extracts—with typical contents of 25% [45]; this can be used directly in the formulation of supplements, or undergo further multistep purification—encompassing hydrolysis to release hydroxylated carotenoids from the accompanying fatty acids, and final recrystallization to polish the product.

Obtaining a carotenoid-rich oleoresin from microalgae—dried or in wet paste form, is a more straightforward task; such extracts may then be subjected to classical processes to obtain purer lutein [45,74] that may successfully compete with that extracted from marigold.

#### 6.2.1. Organic Solvent-Mediated Extraction

Solvent extraction usually resorts to hexane—and has advantages over alkaline treatment because all lutein and zeaxanthin are converted to their free forms, while carboxylic acids and chlorophylls remain in the aqueous phase [45]; this method has been optimized for *S. almeriensis* [74]. Extraction was maximized with a 1:1 (v/v) ratio of hexane to sample, and the optimal number of extraction steps was typically six—which led to 95% recovery of lutein. Less conventional solvents—e.g., ethyl lactate, have been recently proposed [76] for plant matter at large, but can in principle be applied also to microalgae.

A significant improvement would be to eliminate the drying step of microalgal biomass prior to extraction; Fernández-Sevilla *et al*. [77] have accordingly proposed a modification of a previous approach [74] that can handle wet biomass paste (*ca*. 20% DW), based on an extraction phase composed by hexane/ethanol/water and KOH—which simultaneously effects an alkaline treatment to saponify susceptible lipids and extract the intended carotenoids.

Another enhancement is the accelerated solvent extraction methodology, which uses a special type of contactor to circulate solvent at high pressure through a tightly packed bed of biomass. However, high temperatures are required (over 60 °C, and usually as high as 170 °C) to lower the viscosity of the solvent, which leads to formation of pheophorbide from the microalgal chlorophylls that are of a major toxicological concern. In any case, extraction with hexane or ethanol allows easy solvent removal afterwards, as well as high-content lutein extracts [45].

For selective extraction of free astaxanthin from red encysted *Haematococcus* sp., an alternative procedure has been designed that resorts to dodecane and methanol [75]; it consists of dodecane-mediated extraction of the crude mixture, followed by extraction with methanol. The first stage did not require previous cell harvesting, and separation of the dodecane-rich phase from the culture medium containing cell debris proceeded rapidly via plain settling. In the second stage, the free astaxanthin in the former extract was selectively solubilized in methanol along with saponification of astaxanthin esters—thus leading to a total recovery of astaxanthin above 85%.

#### 6.2.2. Green Solvent-Mediated Extraction

An environment-friendly downstream process using common vegetable oils was proposed by Kang *et al*. [79] for direct extraction of astaxanthin from *Haematococcus* sp. As said crude microalgal astaxanthin consists of *ca*. 70% monoesters, 25% diesters and 5% free forms, a rather lipophilic nature results, so vigorous stirring is required to gradually disrupt the cells; the oily extracts are then simply separated from the culture medium containing cell debris by gravity settling. When using olive oil, recoveries of up to 93.9% were possible [79]. Apparently, a similar method had been proposed long before by Nonomura [80], who then claimed up to 7.5% yield of lutein.

#### 6.2.3. Supercritical Fluid-Mediated Extraction

Classical extraction with organic solvents has attained purity degrees sufficient to meet commercial specifications for large-scale production of lutein; however, selective precipitation with supercritical CO_2_ constitutes a promising alternative. Note that conventional liquid extraction of carotenoids from microalgal matrices is time-consuming—as multiple extraction steps are typically required; and large relative ratios of organic solvents have to be used, which are often expensive and potentially harmful. Supercritical fluid extraction (SFE) using modified CO_2_ permits more straightforward purification and shorter extraction times [81].

In general, SFE is relatively rapid and efficient because of the low viscosities and high diffusivities that characterize supercritical fluids. Furthermore, extraction can be made selective by controlling solvent density; the material extracted will be recovered afterwards by simply depressurizing, thus allowing the supercritical fluid to return to its gaseous form and leaving no (or little) residual solvent in the precipitate thus originated [82]. Supercritical CO_2_ has so far been the most employed supercritical fluid—because it is non-flammable, non-toxic, inexpensive and relatively inert from a chemical point of view.

Previous studies demonstrated the feasibility of extracting pigments from plants using supercritical CO_2_—e.g., carotenoids from carrots [83] and cabbages [84]; Mendes *et al*. [85], Careri *et al*. [86] and Macías-Sánchez *et al*. [87–89] have meanwhile extended such a technique to extraction of carotenoids from *C. vulgaris*, *Spirulina platensis*, *Nannochloropsis gaditana*, *Synechococcus* sp. and *S. almeriensis*, respectively—and satisfactory results were consistently reported, as emphasized in Table 3.

However, this mode of extraction tends to recover chlorophylls more efficiently than carotenoids, thus producing extracts with relatively poor specifications [90]. Furthermore, the cost of supercritical fluids and associated equipment make it difficult for SFE to compete with classical solvent extraction—especially because the former requires dry biomass.

The selective adsorption of lutein might constitute an alternative in terms of separation/purification, especially if specific solid phases can be used [91], coupled with contacting conveyed by expanded beds [92]; this allows raw extracts to be processed, and tolerates the presence of cell debris or other particulate matter that causes major problems in conventional preparative chromatography. Selective precipitation was also described by Miguel *et al*. [93], who proposed use of supercritical CO_2_ after organic solvent extraction; the first solvent (containing carotenoids) was accordingly mixed with supercritical CO_2_, and the conditions of pressure and temperature were duly adjusted to promote preferential precipitation of lutein. However, simple standard mixtures—rather than complex microalgal extracts have been considered, so a long way of improvement is still anticipated prior to practical use.

#### 6.2.4. *In Situ* Extraction

*In situ* extraction of β-carotene from *Dunaliella salina* was recently reported by Kleinegris *et al*. [44], using a flat-panel photobioreactor operated as a turbidostat—where the numbers of stressed cells were kept essentially constant via a continuous, well-defined level of irradiation. This two-stage system comprised an organic phase of dodecane, sparged at a rate of 286 L_dodecane_ L_reactor_−1 min^−1^ that promoted formation of an emulsion in the aqueous phase; β-carotene was then continuously extracted from the aqueous to the organic phase, at a rate of *ca*. 2.75 mg_β__-carotene_ L_dodecane_−1 d^−1^ (equivalent to 0.7 mg_β__-carotene_ L_reactor_−1 d^−1^). However, this process exhibited a poor efficiency—as the yield of β-carotene extracted by the solvent was a mere one-tenth of that removed from the reactor via biomass overflow.

If the aforementioned carotenoid-rich biomass was extracted as well, then the overall volumetric productivity of the system would reach 8.3 mg_β__-carotene_ L_reactor_−1 d^−1^; this is still below the yield attained if downstream rather than *in situ* extraction was promoted (*ca*. 13.5 mg_β__-carotene_ L_reactor_−1 d^−1^) [44], so in this system simultaneous biosynthesis and extraction cannot be justified relative to the classical sequential approach.

## 7. Final Considerations

Carotenoid production appears to be one of the most successful case studies of blue biotechnology. The rising market demand for pigments from natural sources has promoted large-scale cultivation of microalgae for synthesis of such compounds, so significant decreases in production costs are expected in coming years.

The recognized therapeutic value of some carotenoids (especially lutein) in prevention and treatment of degenerative diseases has indeed opened new avenues for development of mass production systems. Advances in knowledge of the underlying physiology, biochemistry and molecular genetics of carotenoid-producing microalgae are now urged—which would have a major impact upon development and optimization of this (and alternative) microalga-based technologies. In this regard, the genes encoding enzymes that are directly involved in specific carotenoid syntheses need in particular to be investigated—so that further development of transformation techniques will permit considerable increase of carotenoid cellular contents, and accordingly contribute to increase the volumetric productivities of the associated processes.

## Figures and Tables

**Table 1.** Carotenoids produced by selected microalgae.

**Microalga source**	**Active compound**	**Reference**
*Dunaliella salina*	β-carotene	[13,14]
*Haematococcus pluvialis*	Astaxanthin, cantaxanthin, lutein	[14,18]
*Chlorella vulgaris*	Cantaxanthin, astaxanthin	[14,19]
*Coelastrella striolata* var. *multistriata*	Canthaxanthin, astaxanthin, β-carotene	[40]
*Scenedesmus almeriensis*	Lutein, β-carotene	[41]

**Table 2.** Optimal conditions of production of carotenoids by selected microalgae.

**Carotenoid**	**Microalga source**	**Processing conditions**	**Reactor configuration**	**Productivity**	**Ref**
**β-carotene**	*Dunaliella salina*	**T**: 25 °C; **pH**: 7.5 ± 0.5; **LI**: 281 ± 89 μmol_photon_ m^–2^ s^–1^; **SR**: 38 cms^–1^ s^–1^	Semi-continuous outdoor, closed tubular (55 L)	Biomass: 2 g m^–2^ d^–1^ Total carotenoids: 102.5 ± 33.1 mg m^–2^ d^–1^ (β-carotene: 10% of biomass)	[42]
		**T**: 30 °C; **pH**: 7.5; **LI**: 200–1200 μmol_photon_ m^–2^ s^–1^; **SR:** 0.6 L min^–1^ (N_2_)	Continuous turbidostat, flat-panel (2.5 L)	β-Carotene: 13.5 mg L^–1^ d^–1^ (15.0 pg cell^–1^)	[43]
		**T**: 30 °C; **pH**: 7.5; **LI**: 200–1200 μmol_photon_ m^–2^ s^–1^; **SR:** 0.286 L_solvent_ L^–1^ min^–1^ (organic solvent)	Continuous turbidostat, flat-panel (1.9 L) with *in situ* extraction	β-Carotene: 0.7 mg L^–1^ d^–1^ β-Carotene: 8.3 mg L^–1^ d^–1^ (8.9 pg cell^–1^)	
**Lutein**	*Muriellopsis* sp.	**T**: 28 °C; **pH** 6.5; **LI**: 460 μmol_photon_ m^–2^ s^–1^	Batch (0.2 L, 4–7 d)	Lutein content: 5.5 mg g^–1^ L^–1^ d^–1^ Lutein: 0.8–1.4 mg L^–1^ d^–1^	[44]
		**T**: 28 °C; **pH**: 7; **LI**: continuous 200 μmol_photon_ m^–2^ s^–1^; **AF**: 50–100 L^–1^ h^–1^ (1 %, v/v CO_2_)	Continuous outdoor, tubular (55 L)	Biomass: 7.2 mg L^–1^ d^–1^ Lutein: 5.5 mg g^–1^ L^–1^ d^–1^	[44]
		-	Semicontinuous outdoor, open tank (100 L)	Biomass: 100 mg m^–2^ d^–1^ Lutein: 100 mg g^–1^ L^–1^ d^–1^	[42]
	*Scenedesmus almeriensis*	**T**: 30 °C; **pH**: 8.0; **LI**_max_: 1700 μE m^–2^ s^–1^; **AF**: 0.5 (v/v)/min^–2^ s^–1^; **LDC**: solar cycle	Continuous (2 L)	Lutein: 4.9 mg L^–1^ d^–1^	[8]
		**T**: 35 °C; **LI**: 1900 μE m^–2^ s^–1^	Continuous outdoor, tubular	Lutein: 5.31 mg m^–2^ d ^–1^	[45]
	*Chlorella protothecoides*	**T**: 28 °C; **pH**: 6.5; **LI**: absence of light; **MM**: heterotrophic	Batch (16 L)	Lutein: 10 mg L^–1^ d^–1^	[46]
	*Chlorella zofingiensis*	**T**: 28 °C; **pH**: 7; **LI**: 200 μmol_photon_ m^–2^ s^–1^; **AF**: 50–100 L^–1^ h^–1^ (1%, v/v CO_2_) **LDC**: continuous light	Batch (0.2 L)	Lutein: 3.4 mg L^–1^ d^–1^	[44]
	*Chlorococcum citriforme*			Lutein: 1.05 mg L^–1^ h^–1^	
	*Neospongiococc us gelatinosum*			Lutein: 0.70 mg L^–1^ h^–1^	
**Astaxanthin**	*C. zofingiensis*	**T**: 30 °C; **pH**: 6.5; **LI:** darkness; **SR**: 130 rpm; **MM**: heterothrophic	Batch (250 mL)	Astaxanthin:10.3 mg L^−1^	[47]
	*Haematococcu s pluvialis*	**LI**: day light cycle	Continuous chemostat, tubular (50 L)	Biomass: 0.7 g L^−1^d^−1^ Astaxanthin: 8.0 mg L^−1^d^−1^	[48]
		**T**: 28 °C; **LI**: 345 μmol_photon_ m^−2^ s^−1^	Batch (1 L)	Astaxanthin content: 98 mg g^−1^_biomass_	[49]
		**T**: 15–25 °C; **LI**_max_: 2000 μmol_photon_ m^−2^ s^−1^	Enclosed outdoor (25,000 L)	Biomass: 90 g m^−2^ Astaxanthin: 13 g m^−2^ d^−1^	[39]

**AF**: air flow; **LDC**: light/dark cycle; **LI**: light irradiance; **MM**: metabolic mode; **SR**: stirring rate; **T**: temperature.

**Table 3.** SFE yields of total carotenoids (including lutein), and of lutein specifically, by selected microalgae.

**Microalga source**	**Operating conditions (pressure/temperature/time)**	**Total carotenoids** (mg/g DW biomass)	**Lutein** (mg/g DW biomass)	**Total carotenoids/Chlorophyll*a*ratio**	**Reference**
*Nannochloropsis gaditana*	200 bar/40 °C/180 min	0.152	-	0.524	[87]
	200 bar/50 °C/180 min	0.152	-	0.410	
	200 bar/60 °C/180 min	0.125	-	1.389	
	300 bar/40 °C/180 min	0.208	-	0.258	
	300 bar/50 °C/180 min	0.248	-	0.230	
	300 bar/60 °C/180 min	0.250	-	0.179	
*Chlorella vulgaris*	200 bar/40 °C/198 min	0.011	-	-	[85]
	200 bar/55 °C/180 min	0.008	-	-	
	350 bar/55 °C/252 min	0.080	-	-	
*Synechococcus* sp.	200 bar/40 °C/180 min	0.386	-	193.000	[88]
	200 bar/50 °C/180 min	1.225	-	23.113	
	200 bar/60 °C/180 min	0.405	-	101.25	
	300 bar/40 °C/180 min	0.748	-	32.522	
	300 bar/50 °C/180 min	1.511	-	19.372	
	300 bar/60 °C/180 min	0.808	-	46.316	
*Scenedesmus almeriensis*	200 bar/32 °C/300 min	-	0.0013	-	[89]
	200 bar/46 °C/300 min	-	0.0000	-	
	200 bar/60 °C/300 min	-	0.0109	-	
	300 bar/39 °C/300 min	-	0.0236	-	
	300 bar/53 °C/300 min	-	0.0090	-	
